# A Combination of Cross Correlation and Trend Analyses Reveals that Kawasaki Disease is a Pollen-Induced Delayed-Type Hyper-Sensitivity Disease

**DOI:** 10.3390/ijerph110302628

**Published:** 2014-03-04

**Authors:** Akira Awaya, Chiaki Nishimura

**Affiliations:** 1 Dermatology & Epidemiology Research Institute (DERI), 4978 Totsuka-cho, Totsuka-ku, Yokohama, Kanagawa 244-0003, Japan; 2 Department of Genome System Science, Yokohama City University, Seto 22-2, Kanazawa-ku, Yokohama, Kanagawa 236-0027, Japan; 3 CN Medical Research, Nishiochiai 3-16-15, Shinjyuku-ku, Tokyo 161-0031, Japan

**Keywords:** Kawasaki Disease, pollen-induced disease, cross-correlation, pollen release, pollen exposure, trend, exponential curve

## Abstract

Based on ecological analyses we proposed in 2003 the relation of Kawasaki Disease (KD) onset causing acute febrile systemic vasculitis, and pollen exposure. This study was aimed at investigating the correlation between pollen release and the change in the numbers of KD patients from 1991 to 2002 in Kanagawa, Japan. Short-term changes in the number of KD patients and medium- to long-term trends were analyzed separately. Short-term changes in the number of KD patients showed a significant positive cross correlation (CC) with 9- to 10-month delay following pollen releases, and a smaller but significant CC with 3- to 4-month delay. Further, a temporal relationship revealed by positive CC distribution showed that pollen release preceded KD development, suggesting that pollen release leads to KD development. A trend in patient numbers was fitted by an exponential curve with the time constant of 0.005494. We hypothesized that the trend was caused by the cumulative effects of pollen exposure for elapsed months on patients who may develop KD. By comparing the time constants of fitted exponential curve for each pollen accumulation period with 0.005494, the exposure period was estimated to be 21.4 months, which explains why approximately 50% of patients developed KD within 24 months from birth.

## 1. Introduction

The Tokyo Metropolitan Institute of Public Health has reported that the sum release of Japanese cedar pollens and Japanese cypress pollens in 2010 and 2011 was 1,427/cm^2^ and 15,112/cm^2^ per season [1], respectively. Along with this large increase in pollen release from 2010 to 2011, an increase in the onset of Kawasaki disease (KD) and other diseases was also noted [2,3]. Further, the ratios of patient numbers/sentinel numbers in 2011/2010 for KD, hand, foot and mouth disease (HFMD), erythema infectiosum (EI), and aseptic meningitis were 1.38, 2.25, 1.22, and 2.43, respectively.

We have previously reported that there is a positive correlation between the presence of nevi and resistance to allergic rhinitis/conjunctivitis (pollinosis) [4], and that children of parents who are both or either constitutionally allergic, but do not show conspicuous nevi on the head and neck (another indicator of individuals susceptible to pollinosis), are predisposed to KD [5].

In epidemiological studies, Awaya and Sahashi proposed that KD development was closely related to pollen release; thus, KD may be triggered by pollens [5]. Awaya and Murayama proposed, using regression analyses of the correlation of KD onset and pollen exposure, that KD may be one of a group of pollen-induced delayed-type hypersensitivity diseases [6].

Very recently, Cara *et al.*, referring to a previous paper of Matsuoka *et al.* [7], stated that their report of a child who presented with several clinical features of KD along with mild cardiac echocardiographic alterations, both of which resolved after a diet of protein-free cow’s milk, underscored the relationship between atopy and KD, and that their data supported the hypothesis that the same heritable genetic factors that induced a specific pattern of altered or impaired immune maturation could increase the risk of developing both KD and allergy [8].

The relationship between KD and atopy had been evaluated by Matsuoka *et al.* in a large cohort study that demonstrated a significantly higher incidence of allergic rhinitis and atopic dermatitis in children with KD [7]. In 1997, they proposed that genetic predisposition to atopy could be associated with a higher susceptibility to KD. In addition, patients with KD tend to have a higher risk of developing atopic dermatitis and allergic rhinitis. Furthermore, extremely recently, Woon *et al*., reported on increased risk of atopic dermatitis in preschool children with KD through a population-based study [9].

On the other hand, Frazer recently suggested that the mysterious KD might be transmitted across the Pacific Ocean by air currents high up in the atmosphere [10], mainly referring to the study of Rodo *et al*. [11], and commented that “KD smells like an infectious disease; we’ve just never been able to catch the culprit.” Rodo *et al.* revealed a consistent pattern wherein KD cases are often linked to large-scale wind currents originating in central Asia and traversing the north Pacific Ocean, which supposedly contain causative agents of microbiological origin, supporting the existing conventional concept that KD onset can be attributed to infectious microorganisms [12,13,14,15,16].

In our previous paper [6], regression analysis revealed a positive correlation between the amount of monthly pollen release and the number of KD patients in Kanagawa from January 1991 through December 2002. In this study, this positive correlation was reassessed again multistoriedly with cross-correlation and trend analyses, using exponential function to confirm quantitatively the reproducibility.

## 2. Methods

### 2.1. Data and Sources

We evaluated the number of KD patients (KD Pt. Nos.) in Kanagawa Prefecture, Japan, from 1991 to 2002 as well as the pollen numbers (Po. Nos.) from all species surveyed in Sagamihara City, Kanagawa, from 1991 to 2002. Individual data on KD patients were provided by Professors Yosikazu Nakamura and Hiroshi Yanagawa of the Department of Public Health, Jichi Medical School, Tochigi, Japan. Other data before 1990 and after 2003 have never been given by them. We calculated the date of KD onset for each patient according to the data and summed up a total of 5,917 KD patients, each distributed during every month in the 12-year period. The number of KD patients every month was adjusted according to the demographic data for children in this area. The population variation during the 12 years ranged from 0.96 to 1.01, while that during the first month was 1.00. Po. Nos. (count/cm^2^) have been surveyed daily at the National Hospital Organization SagamiharaNational Hospital (NHOSNH) from 1965, and the data for 12 years were kindly provided by Dr. Hiroshi Yasueda. The pollen species surveyed included the Japanese cedar (*Cryptomeria japonica*), Japanese cypress, Japanese zelkova, ginkgo, saw tooth oak, rice, ragweed, Japanese hop, as well as other identified and unidentified pollens. The total number of pollens from all species was calculated every month.

### 2.2. Data Processing and Analyses

**Figure 1:**
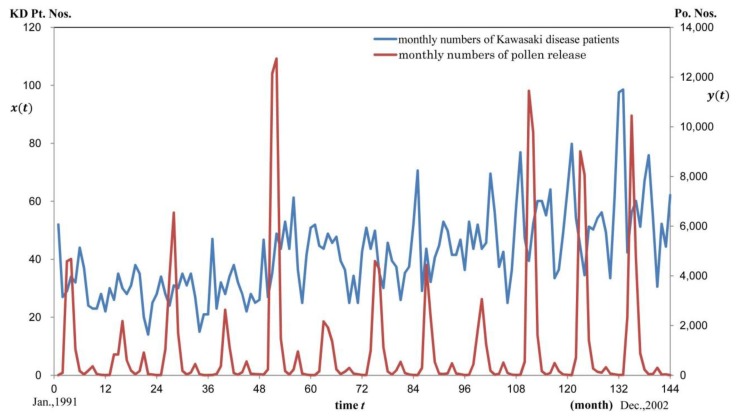
Monthly numbers of KD patients and pollen release during the study period. Population-adjusted monthly KD Pt. Nos. *x*(*t*) and monthly Po. Nos. *y*(*t*) are graphed with respect to time *t* (month). Left axis *x*(*t*) = KD Pt. Nos. Right axis: *y*(*t*) = Po. Nos. Horizontal axis : time *t*. Blue line shows changes in KD Pt. Nos. Red line shows changes in Po. Nos.

In the variation of the population-adjusted KD Pt. Nos. for 12 years (shown in Figure 1 which was made by a different depiction method from that used in Figure 1A of reference [6]), a medium- to long-term element that showed a monotonically increasing trend pattern was seen, as well as a short-term element that showed a fluctuating pattern consisting of a periodic change with about a 1-year cycle and other fluctuations. Therefore, both elements were separately analyzed. For the short-term element, cross-correlation coefficients (ccc) between trend-excluded monthly KD Pt. Nos. and monthly numbers of pollen release in Kanagawa were calculated over the whole period, with the delay time varying from −48 to +48 months. Each ccc was statistically tested with the significance level set at 0.05. The 95% confidence interval was also calculated for each ccc. The trend in KD Pt. Nos. for 12 years was fitted by an exponential function *ae^kt^*, with *a* and *k* being constants, using the least-squares method. Additionally, we speculated that the trend may be caused by the cumulative effect of exposure of patients who may develop KD to pollens during the course of immunological maturation, leading to the development of KD. Hence, we tried to determine the accumulation period of pollens in concordance with the trend in KD Pt. Nos. by applying an exponential function to the integrated amount of Po. Nos. for each accumulation period and comparing its time constant with that of the trend in KD Pt. Nos. An estimate of the accumulation period of pollens was then made using liner interpolation.

### 2.3. Terminology

*t* : time (in months; 1 at January 1991 through 144 at December 2002).

*x*(*t*): population-adjusted numbers of patients with Kawasaki disease (KD Pt. Nos.). “Population-adjusted” is abbreviated in the text.

*y*(*t*): number of pollens (Po. Nos.).

*ae^kt^*: trend factor of *x*(*t*) fitted by exponential function where *a* is a constant and *k* is the time constant; best-fitted at a^*^ = 26.242 and k^*^ = 0.00549.

*w*(*t*): trend-excluded *x*(*t*).

*w*(*t*) = *x*(*t*) - a^*^ *e*^k^*^t^.

*R*(*d*): cross correlation coefficient (ccc) of *w*(*t*) and *y*(*t*) with delay time *d* (-48 ≤ *d* ≤ + 48).


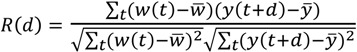
 where *w* and *y* are means of *w*(*t*) and *y*(*t*) over *t*, respectively.

*Z_m_* (*t*): integrated amount of pollens during the preceding *m* months of *t* (9 ≤ *m* ≤ 36).

*Z_m_* (*t*) = *y*(*t*) + *y*(*t* - 1) + ⋯+ *y*(*t* - *m* + 1).

*b_m_ e^j(m)t^*: trend factor of Z_m_(t) fitted by exponential function where b_m_ is a constant and j(m) is the time constant.

## 3. Results

### 3.1. An Analysis of the Cross-correlation between Po. Nos. and Trend-excluded KD Pt. Nos.

The monthly variation in KD Pt. Nos. and Po. Nos. during the 144 months from January 1991 to December 2002 is shown in Figure 1 (see Data Processing and Analyses). Pollen release between February and May was very high, and about 80% of pollens from all species was released in March and April. In addition, a low number of ragweed pollens and rice pollens were released from August to September.

**Figure 2:**
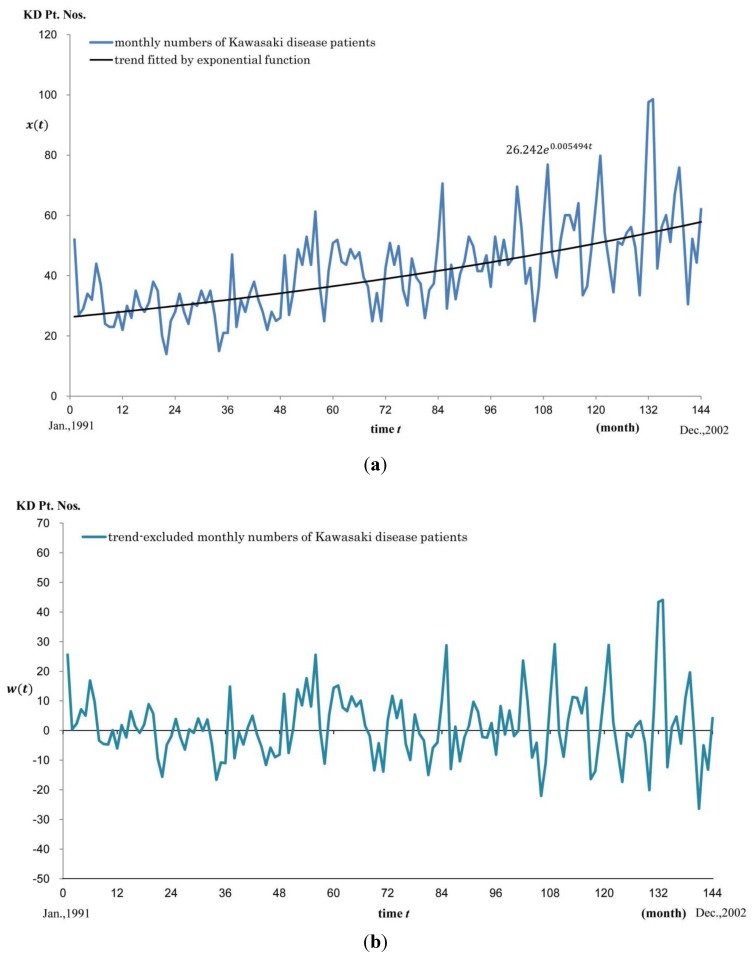
Trends in monthly numbers of Kawasaki disease patients (**a**), and trend-excluded monthly numbers (**b**) during the study period. (**a**) Monthly KD Pt. Nos. *x*(*t*) is graphed with respect to time *t*. Trend line fitted by an exponential function is also shown. Best fitting was given by 26.242*e^0.00549^*. Vertical axis: *x*(*t*) = Monthly KD Pt. Nos. Horizontal axis: time *t*; (**b**) Trend-excluded monthly KD Pt. Nos.*w*(*t*) was calculated and graphed with respect to time. Vertical axis: *w*(*t*) = Trend-excluded monthly KD Pt. Nos. Horizontal axis: time *t*.

The annual KD occurrence patterns were not as regular as pollen release, but certain characteristics were observed during the 12 years, which seemed to consist of 2 or 3 waves: a plateau-like increase starting from March or April and extending to August, followed by a nadir in September or October, and then sharp peaks extending from November to February, which drastically entered into a decreasing phase during February and March. Changes in KD Pt. Nos. as well as its trend line are shown in Figure 2a. The estimated trend line fitted by exponential function *ae^kt^* was 26.242*e^0.005494t^*. As the short-term fluctuating element of the KD Pt. Nos., residual KD Pt. Nos., where the trend was excluded from the original one by subtraction, are shown in Figure 2b.

**Figure 3:**
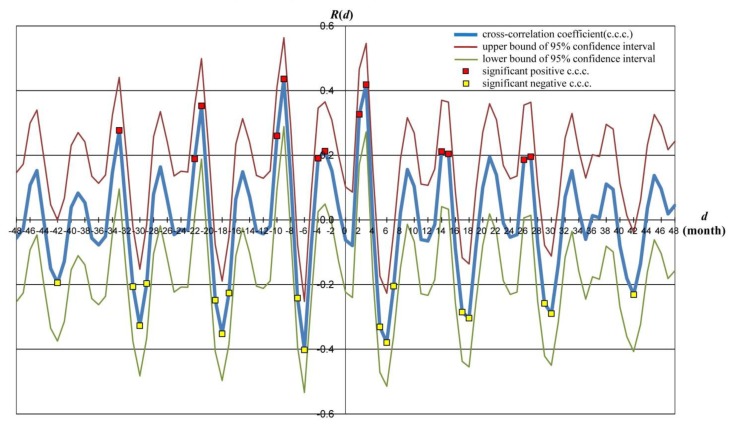
Cross-correlation coefficients (ccc) *R*(*d*) between trend-excluded monthly numbers of KD patients and pollen release during the study period, with delay time *d*. Correlation coefficient *R*(*d*) between *w*(*t*) and *y*(*t + d*) is graphed with respect to delay time *d*. Blue line shows *R*(*d*), and the red and green lines show the upper and lower bounds of the 95% confidential interval of *R*(*d*), respectively. Vertical axis: *R*(*d*) = cross-correlation coefficient. Horizontal axis: delay time *d*.

Then, cross-correlation coefficient *R*(*d*) was calculated between the trend-excluded KD Pt. Nos. and time-shifted Po. Nos. using the delay time dranging from −48 to +48 months, where positive *d* referred to precedence in the time of variation in KD Pt. Nos. to that in Po. Nos. and negative *d* referred to precedence of Po. Nos. to KD Pt. Nos. At the same time, significance of the ccc was statistically tested, and the 95% confidence interval was calculated. Figure 3 shows *R*(*d*) and the confidence interval with its upper and lower bounds for each *d*, with significant positive ccc marked in red, and significant negative ccc in yellow.

As a function of *d*, *R*(*d*) includes three series of peaks within a 12-month period: first, a positive peak series with maximum value at *d* = -9, making an upper envelope of *R*(*d*), which is asymmetrical with respect to *d* = -9, showing a sharp decline in the positive *d* domain; second, a negative peak series with the minimum value at *d* = -6, making a lower envelope of *R*(*d*), which is nearly symmetrical with respect to *d* = -6, with a slight decay to both tails; and third, a positive peak series with maximum but a much smaller value than that in the first series at *d* = -3, and it contains only 1 significant point.

These results show that the pollen release preceded the onset of KD in patients by approximately 9 or 10 months with positive correlation, and the negative correlation series was an inevitable reflection of the 1-year periodicity commonly contained in the variations in KD Pt. Nos. and Po. Nos. In addition, a minor positive correlation with the precedence of pollen release by about 3 months was included in the relationship between the two, which, altogether, strongly suggests that pollen release leads to the development of KD.

### 3.2. Analysis of the Cumulative Exposure of Potential KD Patients to Pollens Using an Exponetial Function

As another additive process that may lead potential patients to develop KD, we assumed that cumulative exposure to pollens for some elapsed time has an effect on the development of KD, which would contribute to the trend-like changes in KD Pt. Nos. On the basis of this assumption, we tried to find the exposure period to pollens that corresponds with the trend in KD Pt. Nos. Hence, a curve *Z_m_*(*t*) of pollens accumulation during the preceding *m* months was plotted, and then the curve was fitted by an exponential function *b_m_ e^j(m)t^*, with *m* varying from 9 to 36.

**Figure 4:**
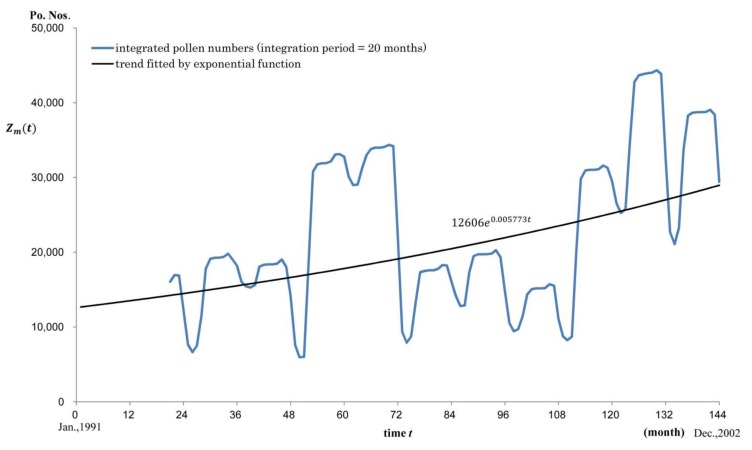
Trend line for the integrated pollen numbers for a fixed integration period (20 months) for each month during the study period *Z_m_*(*t*). is the integrated pollen numbers for *m* months. For example, if *m* = 20, its trend line fitted by exponential function was 12606*e*^0.005773*t*^. Vertical axis: pollen numbers (Po. Nos.). Horizontal axis: time *t*.

**Figure 5:**
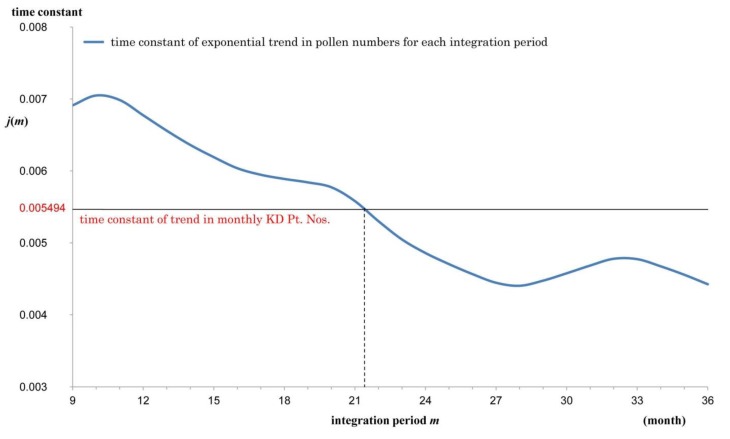
Time constant of the trend for integrated pollen numbers expressed by an exponential function with the integration period as a parameter. The time constant *j*(*m*) is graphed with respect to the integration period *m*. Vertical axis: *j*(*m*) = time constant. Horizontal axis: *m* = pollen integration period. Intersection of the time constant curve of pollens and the constant line of 0.005494 gives an estimate of the pollen integration period in accordance with the trend in KD Pt. Nos. The period was estimated as *m* = 21.4 by linear interpolation.

Figure 4 shows an example where *m* = 20 with the time constant being 0.005773. Figure 5 shows the time constant *j*(*m*) of the fitted exponential curve for each *m*. Comparing *j*(*m*) with 0.005494, the time constant of KD Pt. Nos., the estimated pollen exposure period was 21.4 months.

The KD patients in these 12 years were comprised of patients with KD with 2 types of development: one triggered primarily by transient exposure to a pollen dispersion, with about a 9- to 10-month delay time, and the other triggered by a cumulative exposure to pollens lasting approximately 21.4 consecutive months.

## 4. Discussion and Conclusions

In this study of the monthly variation of the number of KD cases from 1991 to 2002, a short-term trend element that showed a fluctuating pattern consisting of a periodic change with about a 1-year period and other fluctuations was seen and analyzed by cross-correlation analysis. Further, a medium- to long-term element that showed a monotonically increasing trend pattern was seen, and was fitted using an exponential function *ae^kt^* by trend analysis. The time constant *k* was calculated to be 0.0005494. Moreover, we hypothesized that the increasing trend might be attributed to the cumulative effects of pollen exposure during the elapsed months on potential patients toward KD development. By comparing the time constants of the fitted exponential curves for each pollen-accumulation period with 0.005494, the exposure period was estimated to be 21.4 months, which accounts for the fact that about 50% of patients develop KD within 24 months from birth [13]. As a result, positive correlation between Japanese cedar pollen numbers and the development of Kawasaki disease was once again demonstrated [6].

As shown in Figure 1, there were distinct lag time between pollen release and KD onset. It is known that at the time of KD development, inflammation at the Bacille de Calmette et Guérin (BCG) vaccination site (*i.e.*, BCG reactivation such as skin redness, swelling reaction similar to those observed with tuberculin, ulceration, and lesion) is observed in patients who have not undergone skin tests [13,14]. These phenomena suggest us that hyperimmune responses of “delayed-type hypersensitivity” originally triggered by pollen exposure, are increased and propagated in parallel with the involvement of other immune responses. Thus, our study results explain that after sensitization by the first pollen exposure and resensitization with the following pollen exposure, the process leading to the onset of KD, in patients who may develop KD and who are exposed continuously to pollens via the respiratory tract, may progress slowly; finally, in a subacute or chronic manner, KD may develop in infants as a delayed-type hypersensitive reaction different from the conventional delayed-type hypersensitivity such as tuberculin reaction observed in skin tests post BCG vaccination [6].

Figure 3 shows that representatively −10 and −9, and +2 and +3 months in the main sequence of the positive correlation, or −4 and −3 months positive correlation were confirmed between pollen exposure and KD onset. We supposed that −10- and −9-month delays were associated with a high number of KD onsets in December and January, corresponding to the high level of exposure to pollens in March. A lag of +2 and +3 months was assumed because of the relationship between a high number of KD onsets in December and January and the following pollen exposure in March [6]. Further, delays of −4 and −3 months were supposed because of the relationship between considerable amount of KD onsets in June and July and pollen exposure in March.

In our previous regression analysis [6], pollen release during the previous autumn had a comparatively large effect on KD development over several months in such a way that among correlation coefficient (cc) values between Po. Nos. in each 12-month period and KD Pt. Nos. in each 12-month period on the association matrix, mean cc values of Po. Nos. in March were the highest (0.60 ± 0.12), followed by those in October (0.47 ± 0.21). Furthermore, KD onsets in summer (July and August) and winter (January and February) were thought to fall under −10 and −9, and −4 and −3 months from the time of the previous release of Japanese cedar pollen in October [6]. On the other hand, the negative ccc peaks for months with significantly little or no pollen release, such as June, July, and August, may fall under −7 and −6, and +5, +6, and +7 months from KD onsets in winter (December, January, and February).

Next, according to the results from a nationwide survey by a nationwide group investigating KD in Japan, about 80% of patients who developed KD were aged <3 years [13]. Specifically, 25% of patients were aged <1 year, 25% were aged 12–23 months, 18% were aged 2 years, and 12% were aged 3 years [13]. The virtual graphic course curve of chronologically integrated Po. Nos. (as shown in Figure 4) suggests that the newborn infants who may develop KD and who were born at a certain time during 12 years would be repeatedly exposed to pollens for a few months during one spring or multiple spring seasons and multiple times from October to January. They will continue to be sensitized to pollens and the immunological maturation will be enhanced; thus, they will develop systemic vasculitis within 24 months after birth (50%). From our analysis using exponential functions, we were able to quantitatively estimate this time as 21.4 months from the first pollen exposure.

Taken together, the observations of monthly and annual patterns of KD onset and cross-correlation with pollen release may help us to understand how newborns and young children might develop KD each year. Infants with a family history of allergies, who were, for example, born in January or February, may develop KD immediately within a few months if the infants were sensitized with the first pollen exposure during the period of high pollen release (February, March, and April), and were fully immunized with pollens, leading to maturation of the DTH response. Other children who may develop KD may be resensitized with pollen exposure in April and May, or may be led to DTH maturation during the months of June and August, resulting in the development of KD. Infants who did not develop KD during these periods were resensitized due to exposure to a little amount of pollens released from October to January, prior to the seasonal release from February to May, and they developed KD mainly between November and January. Children who may develop KD, but in whom KD did not develop in the first year but developed in the next year instead, may be resensitized with exposure to pollens released in the next spring and autumn season or may be led to DTH maturation, resulting in KD development during spring–summer, or autumn–winter in the next year, respectively.

On the other hand, babies who may develop KD and who were born during May–September, for example, may be sensitized first by exposure to cedar pollens released to a smaller extent from October to January, and then resensitized with pollen exposure mainly in February, March, April, and May, or proceed to be fully immunized in June–September; this leads to development of KD approximately 1 year after birth in about one-fourth of these babies. After 1 year, a similar immunization process is observed in another one-fourth of babies who may develop KD, and maturation of DTH response is observed in the next autumn and spring seasons. Thus, on average, infants who are at risk for KD develop KD 21.4 months after the first pollen exposure.

Since the mid-1980s, various countermeasures to alleviate allergic rhinitis and conjunctivitis (pollinosis) have been being conducted during the spring seasons and in Octobers and Novembers in Japan. Similarly, measures to avoid pollen exposure should be highly recommended for babies who are at risk for KD to protect them from developing serious KD during the first 3 years of life.

As described at the top, Matsuoka *et al.* [7] and Woon *et al.* [9], reported that KD is associated with allergic diseases. Recently, through genetic studies for KD by Onouchi *et al.*, susceptibility genes for KD have been identified in succession with a genome-wide approach, such as functional single-nucleotide polymorphisms (SNPs) in inositol 1,4,5-trisphosphate 3-kinase C (ITPKC) and caspase-3 (CASP3) [17,18]. And Kuo *et al.* reported on polymorphisms of transforming growth factor-β signaling pathway and KD [19]. The results elucidated by Woon *et al*.’s a population-based study in Taiwan that levels of interleukin-5 and IgE were significantly higher in KD patients [9] may be suggestive in relation to our finding of pollen-induced disease of KD. Using the criteria of susceptibility to pollen exposure [4,5], we have been planning earlier to conduct such a population-based study of recent patients whose laboratory data are newly and strictly gathered, and so it is preferable for us to perform such a population-based study in collaboration with not only Japanese but also Taiwanese and Korean groups.

As also described earlier, the number of patients with KD as well as HFMD and EI increased in line with the increase in Po. Nos. It is unclear if all cases of HFMD and EI actually developed due to viral infections caused by parvovirus B19, Coxsackie virus A16, and enterovirus71 [20] in the initial stage of epidemics. Further, whether the seasonal pollen exposure compromises the host’s immune status and how long the compromised status will last remain to be elucidated. Novel animal tests are necessary to elucidate if HFMD and EI may be also pollen-induced delayed-type hypersensitivity diseases in which patients are first sensitized with pollens and then resensitized with continuous pollen exposure during the following years, leading to the onset of these diseases. It is also necessary to study and demonstrate blastoid transformation of lymphocytes sensitized with specific antigenic constituents of pollens, such as Cryj1 and Cryj2 [21], by conducting lymphocyte stimulation tests [22,23] in KD patients.

Furthermore, it is important to conduct clinical epidemiological examinations to determine whether infants with KD develop HFMD or EI later during childhood, or if children with HFMD or EI develop KD later. Future researches should be directed towards studying whether these 3 illnesses develop at the same time in a child, or separately during the course of childhood. Results of these studies will help in enhancing our knowledge of pollen-induced diseases.

*Objective*: To investigate the relationship between the incidence of Kawasaki Disease and pollen release, changes in the number of KD patients from 1991 to 2002 in Kanagawa, Japan were examined by means of the cross-correlation. *Setting*: In 2011, 12,774 people in Japan developed KD, which results in acute febrile systemic vasculitis. The incidence of KD in Japan was found to increase along with the large increase in the amount of pollen released in 2010–2011. *Methods*: Short-term changes in monthly numbers of KD patients of total 5917 and medium- to long-term trends were analyzed separately using cross-correlation (CC) analysis and trend analysis. *Results*: Short-term changes in the number of KD patients showed a significant positive CC with 9- to 10-months’ delay following pollen releases, and a smaller but significant CC with 3- to 4-months’ delay. A temporal relation revealed by positive CC distribution also showed that pollen release preceded KD development, strongly suggesting that pollen release leads to KD development. The trend in patient numbers was fitted by an exponential curve, in the trend analysis, with the time constant of 0.005494. We hypothesized that the trend was caused by the cumulative effects of pollen exposure for the elapsed months on patients who may develop KD. *Conclusions*: By comparing the time constants of the fitted exponential curves for each pollen accumulation period with 0.005494, the exposure period was estimated to be 21.4 months, which explains why approximately 50% of patients developed KD within 24 months of birth.
